# Root canal re-treatment with gutta percha - which techniques influence success?

**DOI:** 10.1038/s41432-024-01019-1

**Published:** 2024-05-25

**Authors:** Alexander Hall, Emilie Baerts, David Edwards

**Affiliations:** 1 Newcastle upon Tyne Hospital NHS Foundation Trust, Newcastle upon Tyne, NE2 4AZ UK; 2 School of Dental Sciences, Newcastle University, Framlington Place, Newcastle upon Tyne, NE2 4BW UK

**Keywords:** Root canal treatment, Endodontic files

## Abstract

**Objective:**

A systematic review and meta-analysis of the literature was carried out assessing the success rate of root canal retreatment using gutta percha.

**Data sources:**

Four of the largest databases were used to identify existing literature with no date or language restrictions. PubMed, Cochrane, ScienceDirect, Scopus and other additional sources were searched. Grey literature was also reviewed.

**Study selection:**

Two authors, with Master’s degrees in endodontics and with extensive university teaching experience, were selected to screen the databases to identify suitable studies. In case the authors were not able to agree during the study selection process, a third investigator was consulted. Specific inclusion and exclusion criteria were outlined and adhered to in the study selection. Two randomised controlled trials, seven single arm prospective studies and one single arm ambispective study published before the 10th of December 2022 were included. These studies evaluated the success of root canal re-treatment, obturated with gutta percha with at least a 1-year follow-up. Nine of the studies were published between 1998 and 2022. Seven studies were conducted in Europe, one in North America and one in Asia.

**Data extraction and synthesis:**

Standard Cochrane methods to assess interval validity were used. Risk of bias in individual studies was assessed using The Newcastle-Ottawa quality assessment scale (NOS) for single-arm studies, and the Cochrane risk of bias tool (RoB2) was used for randomised controlled trials. Outcome measures were standardised as either success or failure of root canal retreatment. Success was classified into 2 different criteria: Strict criteria = absence of clinical signs and symptoms and radiographically normal periodontal ligament space; and Loose criteria = absence of clinical signs and symptoms and absence or reduction of apical radiolucency in the control radiograph. Statistical analysis was undertaken using R software and the Freeman-Turkey transformation was performed. Results were visualised using forest plots. Heterogeneity between studies was measured using the Cochrane Q test and I^2^ values.

**Results:**

Whilst following strict criteria, the success rate of non-surgical root canal retreatment obturated with gutta percha was 71% for 1–3 years follow-up (95% CI, 0.66–0.77) and 77% for 4–5 years follow-up (95% CI, 0.67–0.86). Heterogeneity was moderate (I^2^ = 61.4) and low (I^2^ = 0.0), respectively. Factors reducing the success rate of root canal re-retreatment under the strict criteria were older patients, mandibular teeth, molar teeth, the presence of a peri-apical radiolucency, teeth with a previous radiolucency, large peri-apical radiolucency’s, higher initial periapical index scores and multiple visit-retreatments. Following the loose criteria, the success rate of non-surgical root canal re-treatment obturated with gutta percha was 87% for 1–3 years follow-up (95% CI, 0.79–0.93) with significant heterogeneity across the studies (I^2^ = 88.5%). Factors influencing the success rate under the loose criteria were large periapical lesions >5 mm and higher initial periapical index (PAI) scores.

**Conclusions:**

Non-surgical root canal retreatment results in favourable outcomes. However, there are several factors which can result in a lower success rate: the presence and size of a periapical radiolucency, a higher initial PAI score, multiple-visit retreatments, and the size and position of the tooth.

## A Commentary on

**Olivieri J G, Encinas M, Nathani T, Miró Q, Duran-Sindreu F**.

Outcome of root canal retreatment filled with gutta-percha techniques: a systematic review and meta-analysis. *J Dent* 2024; **142**: 104809.

**GRADE rating:** 

## Commentary

Root canal treatment is defined as the combination of mechanical instrumentation of root canal system, its chemical debridement and filling with an inert material, designed to maintain or restore the health of the peri radicular tissues in order for the tooth to be able to function for as long as possible^1^.

Root canal treatment may fail in approximately 15–25% of cases^2^. The usual factors which can be attributed to endodontic failure are persistence of bacteria, inadequate filling of the canal, overextension of root filling materials, improper coronal seal, untreated canals, iatrogenic procedural errors and complications of instrumentation^2^.

Where a patient wishes to retain a tooth, the treatment options for root canal treatment include surgical or non-surgical root canal re-treatment. It is recommended to use a non-surgical approach when the root canal anatomy is unaltered, and when the cause of failure can be overcome^3^.

The main aim of this systematic review and meta-analysis was to evaluate the success of root canal re-treatment obturated with gutta percha^4^. The secondary aim was to analyse the variables to identify possible associations with treatment outcomes. The review was registered with the International Prospective Register of Systematic Reviews (PROSPERO) ensuring transparency and minimising potential bias and unplanned review duplication^5^.

Although the review did not clearly display a PICO (Population, Intervention, Comparator, Outcome) question, the outcomes considered are clear, distinctly defining strict and loose inclusion criteria. This has relevance to dental patient related outcome measures (dPROMs), which measure subjective elements of conditions and evaluate the burden of treatment and disease from a patients’ perspective^6^. Over the past two decades, the use of dPROMs has significantly increased, becoming more important in modern healthcare.

A risk of bias table was set up using the Newcastle-Ottawa quality assessment scale (NOS) modified for single group cohort studies and Cochrane Collaboration’s tool for randomised controlled trials. All studies were assessed as having an overall low risk of bias. Generally, the internal validity of included studies was good, with high quality studies of low risk of bias being included.

The authors searched for a range of study designs without date or language restrictions. Grey literature was also included. There was a robust search strategy using four of the largest databases and standard systematic review methodology including involvement of a third reviewer to resolve disagreement in inclusion. The inclusion criteria were clearly reported and appropriate, including clinical trials, randomised control trials, case-control studies, cross-sectional studies and cohort studies. By encompassing a wide range of study type, the often-limited conclusions of systematic reviews that only include randomised controls has been avoided, maximising the findings and impact of this research. Furthermore, included studies were found to be high quality with a low-dropout rate.

The root canal re-treatment was carried out by endodontic post-graduate students in 3 of the studies, by specialists in the field in 4 studies and by both in 1 study. In 2 studies, it was not recorded who carried out the treatment. In addition, only 2 of the studies were carried out in the UK. The other 8 were carried out in Europe, meaning that most of the included studies are European. These factors may limit the external validity of the study to UK general dental practitioners working in primary care. The included papers suggest that root canal re-treatment offers a predictable treatment option for managing failed primary endodontics when undertaken by a specialist or by a dentist with enhanced skill.

When evaluating success under strict criteria, the meta-analysis showed single-visit retreatments had a higher success rate than multiple-visit retreatments. Root canal treatment can be either completed with a single visit approach, which involves obturating straight after instrumentation and irrigation; or in a multi-visit approach, in which two or more sessions are involved with the obturation performed in the final session. According to a paper by Wong 2014^7^, it shows there is currently no evidence showing that one treatment regimen is more effective than another. Although, a higher proportion of patient’s who underwent single visit RoCT reported pain within one week compared to those who underwent multiple visit RoCT.

Endodontic disease can be commonly asymptomatic therefore radiological assessment is required. Apical periodontitis usually appears as a periapical radiolucency on radiographs. This is caused by reduced mineral density of the affected bone in response to localiSed inflammation, mostly caused by microbial infection within the root canal system. The absence of a periapical radiolucency is a key determinant of treatment success^8^. Recently, survival of endodontic treatment has become another outcome measure- including teeth that have radiographical signs of periapical pathology but are judged as acceptable by patients. This systematic review has only looked at success, not survival criteria.

In summary, this was a high-quality paper including several well selected and strong research papers on the topic. It shows that non-surgical root canal retreatment generally results in favourable outcomes. The most significant factors causing a lower success rate were: a higher PAI score and the presence and size of a periapical radiolucency. However, the relevance to primary care can be questioned as most papers included involved treatment carried out by endodontic specialists. Further research into the topic could include another systematic review, including papers where treatment has been carried out by general dental practitioners.

Practice points

- Larger periapical radiolucencies decrease the success of endodontic retreatment with gutta percha.
- Consider carrying out root canal retreatment over multiple visits to reduce post-operative pain.
